# Adverse effects of paternal chemotherapy exposure on the progeny brain: intergenerational chemobrain

**DOI:** 10.18632/oncotarget.24311

**Published:** 2018-01-23

**Authors:** Anna Kovalchuk, Yaroslav Ilnytskyy, Rafal Woycicki, Rocio Rodriguez-Juarez, Gerlinde A.S. Metz, Olga Kovalchuk

**Affiliations:** ^1^ Canadian Center for Behavioural Neuroscience, Department of Neuroscience, University of Lethbridge, Lethbridge, AB, T1K3M4, Canada; ^2^ Department of Biology, University of Lethbridge, Lethbridge, AB, T1K 3M4, Canada; ^3^ Alberta Epigenetics Network, Calgary, AB, T2L 2A6, Canada

**Keywords:** chemotherapy, brain, transcriptome, epigenetics, transgeneration effects

## Abstract

Recent advances in cancer treatments have led to significant increases in cure rates. Most cancer patients are treated with various cytotoxic chemotherapy regimens. These treatment modalities are mutagenic and genotoxic and cause a wide array of late-occurring health problems, and even exert a deleterious influence on future offspring. The adverse effects from exposed parents on offspring are referred to as transgenerational effects, and currently little is known about chemotherapy-induced transgenerational effects. Furthermore, transgenerational effects have not been studied in the brains of progeny of exposed parents. In this study, we analyzed the existence and molecular nature of transgenerational effects in the brains of progeny of animals exposed to three common chemotherapy agents: cyclophosphamide (CPP), procarbazine (PCB) and mitomycin C (MMC). For the first time, our results show that paternal exposure to chemotherapy drugs causes transgenerational changes in the brain of unexposed progeny. Although no DNA damage was observed in terms of γH2AX levels, some alterations were found in levels of PCNA, protein involved in DNA repair, replication and profileration. Furthermore, there were changes in proliferation and apoptosis proteins BCL2 and AKT1, the proteins associated with DNA methylation, DNMT1 and MeCP2. Some altered expression trends were noted in proteins involved in myelin biogenesis, MBP and MYT1L. Moreover, global transcriptome profiling revealed changes in over 200 genes in the whole brains of progeny of animals exposed to CPP, and the changes in the levels of FOXP2 and ELK1proteins were confirmed by western blot analysis. These findings suggest that paternal chemotherapy significantly affects offspring brain development and may affect brain functioning. This research provides a key roadmap for future investigations of the novel phenomenon of transgenerational effects of chemotherapy in the brain of progeny of exposed parents.

## INTRODUCTION

Cancer is the leading cause of death in Canada. In addition, in Canada today, cancer is the leading cause of death in children and young adults, and currently one in 400 Canadian adults are survivors of childhood cancer [1, 2]. The number of new cases in Alberta alone is expected to double to more than 26,000 cases per year by 2025 [3]. In recent decades, advances in treatment have led to significant increases in cancer survival and cure rates. In the United States, the five-year survival rate (1985–1999) for children diagnosed with cancer was 75.8% [4]. For Americans aged 20 to 39, the prevalence of childhood cancer survivors is approximately 1 in 640 [5]. Most cancer patients are treated with various cytotoxic chemotherapy regimens [6, 7]. These treatment modalities are mutagenic and genotoxic; therefore, more than two-thirds of cancer survivors experience a wide array of late-occurring health problems [1, 2, 6–8].

Additionally, for cancer patients, their ability to have healthy children is of great importance, especially due to the fact that it is now well-accepted that parental exposure to various environmental stressors can exert negative influences on future offspring. These adverse effects in the offspring of exposed parents are referred to as transgenerational or intergenerational effects [9, 10].

There is a wealth of evidence that radiation exposure effects can span several generations, and leads to transgenerational effects in the offspring of exposed parents [11–13]. While transgenerational effects have been reported in exposed human populations including atomic bomb survivors, individuals affected by the Chernobyl accident, and those living near nuclear test sites [14], their nature and magnitude in the exposed human population remains controversial [15, 16]. Nevertheless, they are also well-documented in rodents, and rodent models are accepted as valuable, albeit not ideal, tools for transgenerational effect analysis, especially for transgenerational carcinogenesis [11, 17–19]. Furthermore, the observed radiation-induced transgeneration effects are primarily paternal in nature [11, 20], with irradiation manifesting both phenotypically and at the genome level in affected progeny. Phenotypic effects include decreased fertility, teratogenic effects and an increased predisposition to cancer [18, 21, 22]. Genome alterations in the progeny of exposed parents appear as increased levels of DNA damage and mutation rates, as well as elevated frequencies of chromosome aberrations and micronuclei [11, 18, 23]. Furthermore, progeny of exposed parents also exhibit altered gene expression patterns and epigenetic changes [24, 25].

While the initial data on transgenerational effects have been obtained from analysis of the progeny of radiation-exposed parents, further studies have revealed that a wide variety of other genotoxic agents induce transgenerational effects [6, 17, 26], such as pesticides and air pollution [27]. Also, there is strong evidence that parental exposure to anticancer chemotherapy drugs can cause heritable transgenerational effects [6, 7]. The pioneering works from the Dubrova laboratory revealed that paternal exposure to clinically relevant doses of bleomycin, cyclophosphamide, and mitomycin C lead to statistically significant and dose-dependent increases in mutation rates in the germline of treated male mice and caused transgenerational genome instability in the offspring of exposed animals [6, 7].

While it has been established that transgenerational effects exist, the exact molecular mechanisms of these phenomena have yet to be defined. Furthermore, recent evidence suggests that transgenerational effects may be epigenetic in nature [10, 18, 28] and are associated with gene expression changes. In addition, the existence of transgenerational effects has been established in organs that are known targets of blood malignancies, such as the spleen, thymus and liver, as well as the white blood cells [24]. This is primarily due to the fact that transgenerational radiation effects often manifest themselves as an increased predisposition to blood cancer. Furthermore, several transgenerational effects of social and psychological stressors have been shown to affect the brain of their offspring [29, 30]. Nevertheless, currently nothing is known about the effects of paternal exposure to chemotherapy drugs on the brain of their unexposed offspring.

Here we set out to analyze molecular gene expression signatures and protein expression profiles in the brains of offspring from males that were exposed to three widely-used cytotoxic chemotherapy drugs, cyclophosphamide (CPP), procarbazine (PCB) and mitomycin C (MCC), and prior to conception. We hypothesized that paternal exposure to these cytotoxic drugs may affect gene expression and protein levels in the brain of unexposed offspring.

## RESULTS AND DISCUSSION

### Paternal chemotherapy altered levels of KU70 and PCNA in the progeny

Histone H2AX is rapidly phosphorylated at Ser139 upon induction of DNA strand breaks, and it can be effectively detected using specific antibodies [31]. Analysis of histone H2AX phosphorylation (levels of γH2AX) is widely used to assess the extent of damage to cellular DNA [31]. The induction of DNA damage and H2AX phosphorylation constitute well-established transgenerational effects. Therefore, the levels of γH2AX in frontal cortex (FC) and brain (BR) tissues of the progeny of control and exposed animals were analyzed.

Surprisingly, no H2AX phosphorylation was detected in any tissue in any of the three independent iterations of the experiment (data not shown). This may indicate that there was no DNA damage induced in the brain tissues of progeny by the pre-conception paternal exposure to chemotherapy agents. Moreover, the brain is highly plastic and has a huge capacity to repair DNA damage and maintain DNA integrity. Additional studies would be needed to analyze the levels of DNA damage in the brain of progeny of exposed parents using other methods.

**Figure 1:**
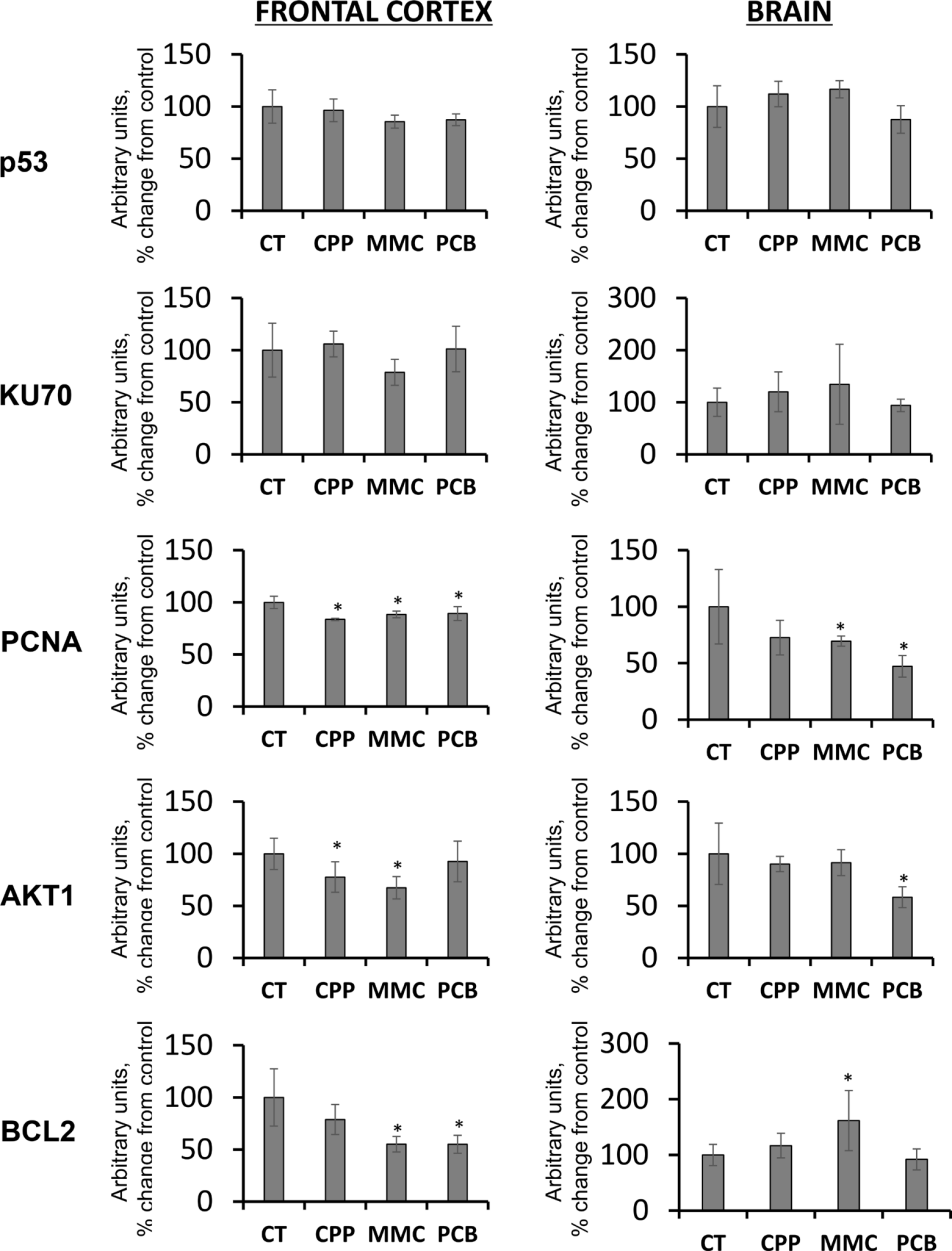
Changes in the levels of DNA damage-, DNA repair- and apoptosis-related proteins in the frontal cortex (FC) and whole brain (BR) tissues of progeny of chemotherapy-exposed parents CPP – cyclophosphamide; MMC – mitomycin C; PCB – procarbazine. Data are shown as average (with SD) arbitrary units of intensity calculated from six individual data points per each experimental group. Due to protein size differences and scarcity of tissue, membranes were re-used several times. Each data point was related to an average intensity of actin samples in a corresponding group and expressed as percent change from controls. sterisks (^*^)indicate statistically significant (*p* < 0.05) difference to unexposed control Student's *t*-test.

Analysis of p53, a key cellular gatekeeper involved in orchestrating DNA damage responses, cell cycle control, and apoptosis [32, 33], did not reveal any significant changes in its levels in either FC or BR samples (Figure 1). No significant changes were seen in the levels of KU70, a DNA repair protein which participates in the non-homologous end-joining mechanism of DNA repair [34], albeit some insignificant trends were noted (Figure 1).

Contrarily, the level of another protein, the proliferating cells nuclear antigen (PCNA) involved in DNA replication and repair DNA synthesis [34, 35], was statistically significantly (*p <* 0.05) substantially reduced in all frontal cortex samples in response to all treatments, and in the whole brain samples in response paternal MMC and PCB exposures (Figure 1). The observed decrease in the levels of PCNA may indicate alterations in the levels of cellular growth and proliferation.

### Paternal chemotherapy changed levels of BCL2 and AKT1 in the progeny

DNA damage and repair are closely linked to the control of the cell cycle and apoptosis. To repair DNA damage, cells need time, which is gained by implementing cell cycle arrest. In the case of inefficient or delayed repair of DNA damage, cells undergo apoptosis.

Although no direct evidence of DNA damage in the brain tissues of progeny of exposed parents was observed, this study proceeded to analyze the levels of several key proteins involved in cell cycle control and apoptosis. Among these, the levels of cellular anti-apoptotic protein BCL2 [36] was analyzed. A significant decrease in BCL2 levels was found in the FC of progeny of animals exposed to all MMC and PCB chemotherapy drugs (Figure 1). A decrease in the levels of anti-apoptotic BCL2 may lead to elevated levels of apoptosis in the FC of progeny. Moreover, FC samples also exhibited reduced levels of PCNA.

In the BR, contrarily, paternal exposure to MMC led to an increase in BCL2 levels (Figure 1). Such an increase may result in a reduction in the levels of apoptosis in brain regions other than the FC. In the future, it would be important to analyze the levels of other apoptosis-related proteins in the brains of progeny of chemotherapy-exposed parents. It would also be prudent to conduct staining to reveal apoptotic cells in order to determine if the levels of apoptosis were indeed affected and, if so, in which brain region cell types this occurred.

Also noted was a decrease in the levels of AKT1 in the FC of progeny of fathers exposed to CPP and MMC, and in the whole brains (BRs) of progeny of PCB-exposed fathers (Figure 1). AKT1 is important in controlling organismal growth and development, and AKT1-deficient mice display impaired overall growth [37]. Moreover, AKT1 plays a key part in brain development and neuronal survival [37, 38]. Therefore, a reduction in AKT1 levels may be a concern that deserves future attention, especially when it is parallel with a reduction in PCNA levels and decreased levels of anti-apoptotic BCL2 proteins. A reduction of AKT1 levels in very young animals may negatively affect brain growth and development and may, therefore, have detrimental consequences for progeny of chemotherapy-exposed parents. More studies would be needed in the future to understand the roles of AKT1 and AKT1-associated signaling in transgenerational changes in the brain.

### Exposure to MMC and PCB Affect DNMT1 and MeCP2 levels

**Figure 2:**
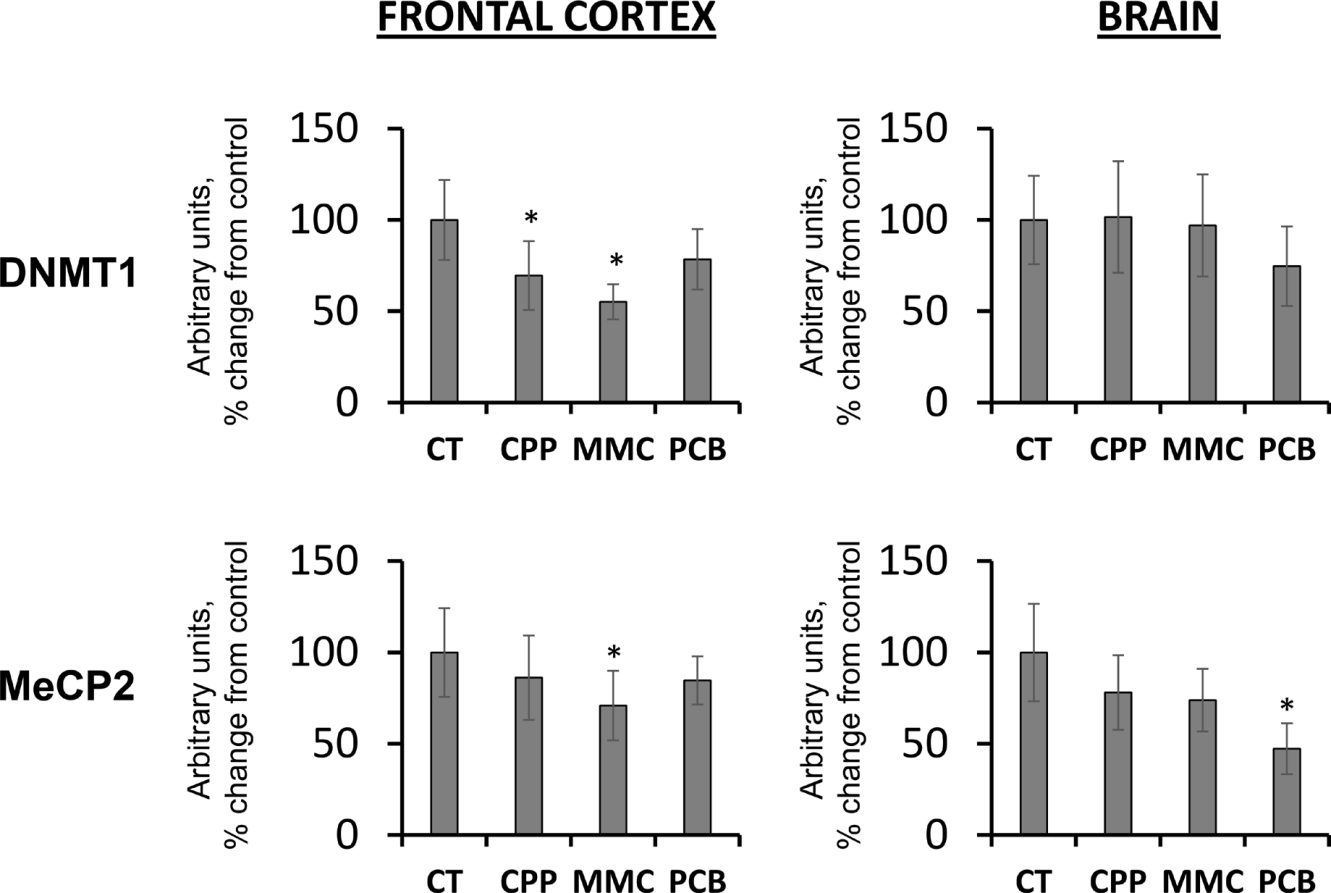
Changes in the levels of DNMT1 and MeCP2 in the brain tissues of progeny of chemotherapy-exposed parents CPP – cyclophosphamide; MMC – mitomycin C; PCB – procarbazine. Data are shown as average (with SD) arbitrary units of intensity calculated from six individual data points per each experimental group. Due to protein size differences and scarcity of tissue, membranes were re-used several times. Each data point was related to an average intensity of actin samples in a corresponding group and expressed as percent change from controls. Asterisks (^*^) indicate statistically significant (*p* < 0.05) difference to unexposed control, Student's *t*-test.

Gene expression is controlled by DNA methylation. DNA methyltransferases regulate methylation patterns in the genome. DNMT1 functions as a maintenance methyltransferase and is responsible for maintaining the methylation patterns upon replication [39]. Changes in its expression may, in turn, influence DNA methylation levels. The DNMT1 level was found to be significantly reduced in the FC samples of progeny of CPP- and MMC-exposed fathers (*p <* 0.05) (Figure 2). No significant changes were seen in the BR samples. Similar trends were found in the levels of the methyl-CpG binding protein, MeCP2 (Figure 2), which binds to methylated DNA in order to stabilize DNMT1 functions and induce chromatin remodeling and gene silencing [39]. The loss of MeCP2 may be associated with genome instability. Furthermore, previous studies have shown a decrease in the levels of MeCP2 in progeny of radiation-exposed parents [24]. Additionally, MeCP2 is critical for establishing and maintaining neuronal networks and brain anatomy [40, 41]. Therefore, more studies are needed to dissect the biological and functional repercussions of the observed loss of MeCP2 levels in the brains of progeny of chemotherapy-exposed parents.

### Myelin transcription factor MYT1L and myelin basic protein levels change in progeny of animals exposed to MMC and PCB

Maternal stress can influence proteins involved in the etiology and pathogenesis of neurological diseases [42]. With this in mind, the levels of myelin basic protein (MBP) and the myelin transcription factor, MYT1L, in the brains of progeny of chemotherapy-exposed parents were analyzed. MYT1L is involved in regulating the development of the nervous system [43, 44]. It is one of three factors that are sufficient to convert mouse embryonic and postnatal fibroblasts into functional neurons. MBP is important in the process of myelination of the nerves in the nervous system [45].

**Figure 3:**
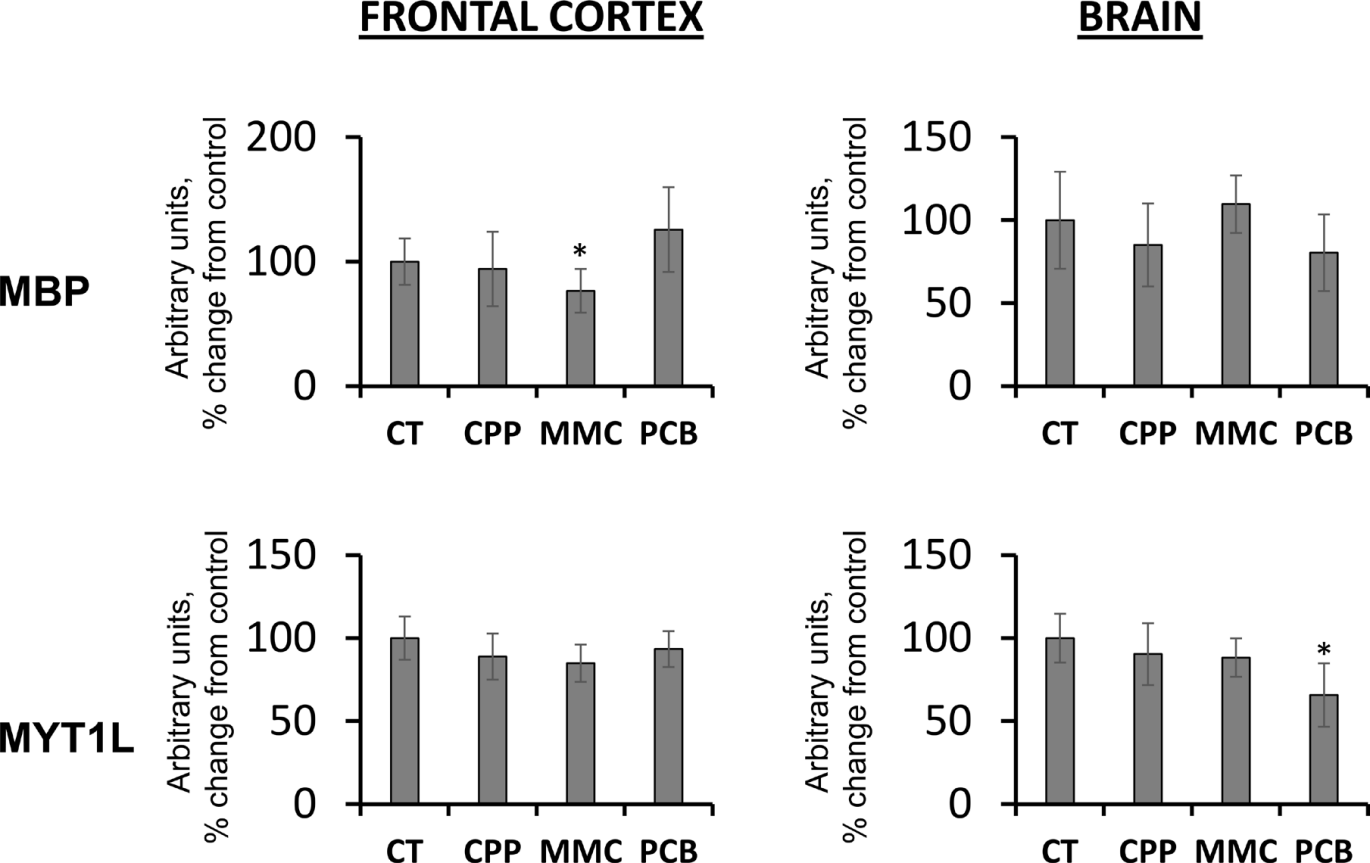
Levels of MBP and MYT1L in the brain tissues of progeny of chemotherapy-exposed parents CPP – cyclophosphamide; MMC – mitomycin C; PCB – procarbazine. Data are shown as average (with SD) arbitrary units of intensity calculated from six individual data points per each experimental group. Due to protein size differences and scarcity of tissue, membranes were re-used several times. Each data point was related to an average intensity of actin samples in a corresponding group and expressed as percent change from controls. Asterisks (^*^) indicate statistically significant (*p* < 0.05) difference to unexposed control, Student’s *t*-test.

It was noted that paternal exposure to MMC resulted in decreased levels of in the FC of unexposed progeny of chemotherapy-exposed parents, whereas paternal exposure to PCB resulted in decrease of MYT1L in the BR samples of progeny (Figure 3). No difference was observed in response to paternal CPP exposure. Recent studies have suggested that MYT1L may be a candidate gene for intellectual disability (ID) [44]. Therefore, in the future, it would be important to further analyze the levels of MYT1L and its role in the brain of progeny of exposed parents. MBP levels need further investigation, since the current protein level trends are inconclusive.

In general, very little is known about the effects of MCC and PCB on the nervous system of exposed animals as well as on the nervous system of progeny of exposed animals. In contrast, there is a substantial body of evidence about the effects of CPP. Transient hippocampal-based memory deficits following a single injection of CPP were found in two independent mouse model-based studies [46, 47]. Also, CPP-treated rats showed significantly impaired behavior, namely poor performance on the novel place recognition task and the contextual fear conditioning task, which was likely due to a disruption in the hippocampal-based memory function [48]. These CPP-treated animals also had a significant reduction in the number of mature neurons, suggesting impaired neurogenesis.

Furthermore, Hsu et al. (1987) showed that paternal CPP treatment resulted in significant changes in several neurotransmitter enzymes in various brain regions of the immediate progeny [49]. Also, the F1 progeny of male rats chronically exposed to CPP exhibited behavior deficits when compared to controls [50, 51]. At that time, the authors believed that this was likely due to genetic effects induced by CPP exposure.

In contrast, the results of Lyons et al. (2011) indicate that CPP acutely reduces the survival of newly-born hippocampal cells, but does not have any long-term effects on spatial working memory or hippocampal proliferation [52]. Examination of the offspring of treated animals showed an increased post-natal mortality rate, paralleled by diminished learning capacity and reduced spontaneous activity in the adults. These changes also persisted in the second generation of exposed animals. Analyses of the brains of the first and second generations showed diminished activity of hippocampal choline acetyltransferase, as well as a decreased level of norepinephrine in the fronto-parietal cortex in the second generation [52].

### Global genome expression analysis revealed substantial changes in the whole brain of the progeny in response to paternal CPP exposure

To further investigate changes in the gene expression in progeny of animals exposed to chemotherapy drugs, whole transcriptome analysis in the FC and BR samples was performed. After averaging the data for five animals per group, significant differences were only found in the BR samples of CPP-exposed animals. There were 137 up- and 131 down-regulated genes. After the −0.3 > log2 > 0.3 cut-off was implemented, 86 up- and 75 down-regulated genes were found.

#### Reactome analysis

**Figure 4:**
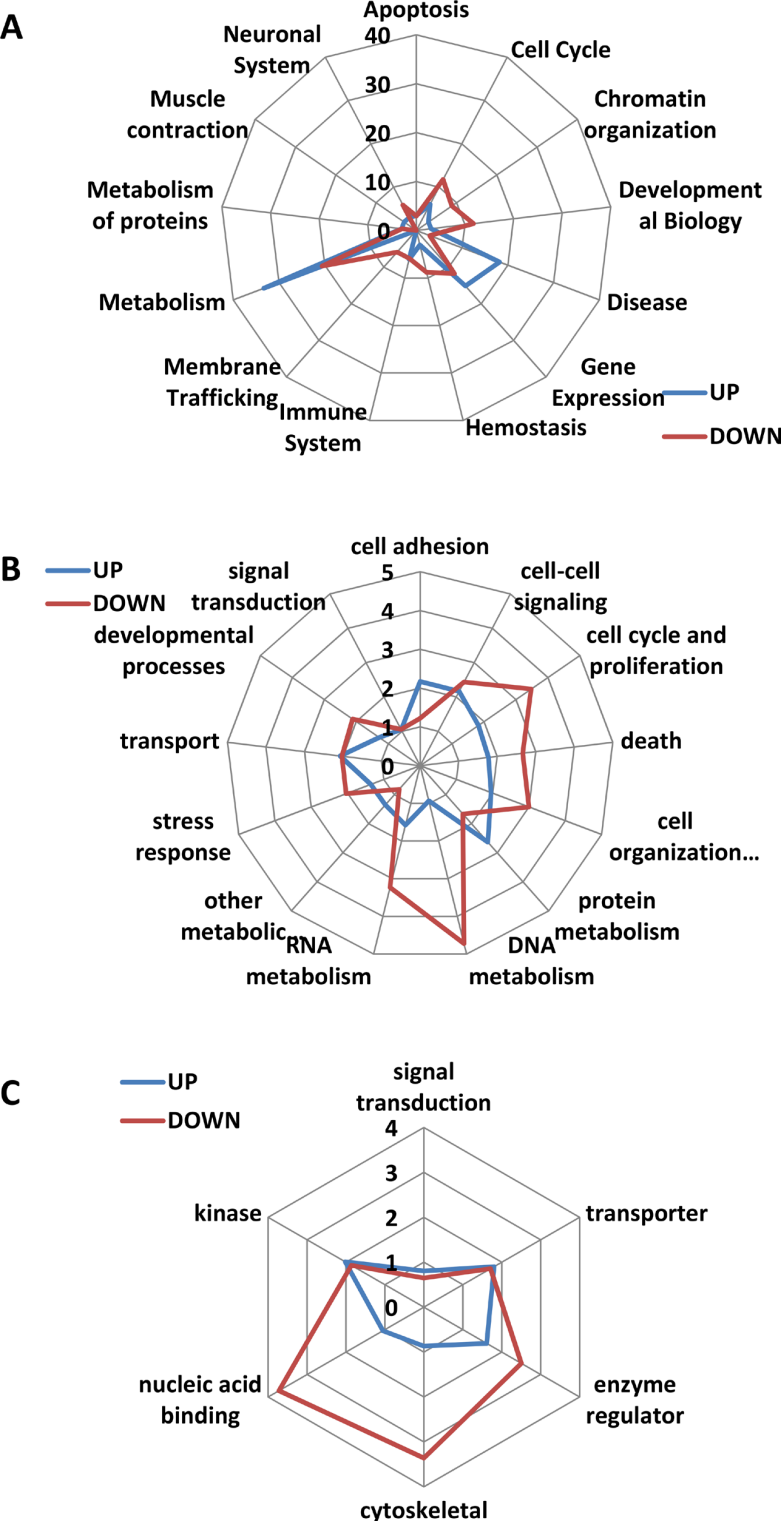
Reactome analysis and analysis of GO terms for biological processes and molecular functions (**A**) Reactome analysis. Reactome web graphic shows the percentage of up- and down-regulated genes belonging to a particular reactome out of all genes for which reactome data were found. (**B**) GO terms enrichment analysis for biological processes. Graphic shows the percentage of up- and down-regulated genes belonging to a particular reactome out of all genes for which GO terms for biological processes were found. (**C**) GO terms enrichment analysis for molecular functions. Graphic shows the percentage of up- and down-regulated genes belonging to a particular reactome out of all genes for which GO terms for molecular functions were found.

The information about enriched pathways for reactome analysis was found for 33 up- and 34 down-regulated genes. The analysis showed enrichment in up-regulated genes belonging to “disease”, “gene expression” and “metabolism” reactomes, and in down-regulated genes belonging to “metabolism”, “cell cycle”, “developmental biology” and “gene expression” reactomes (Figure 4A). Genes belonging to “disease”, “metabolism” and “gene expression” reactomes were common in the up- rather than the down-regulated gene group. In contrast, genes belonging to “cell cycle” and “chromatin organization” reactomes were more common in the down-regulated gene group.

#### Gene ontology analysis

Next, functional annotation analysis was performed using DAVID’s Functional Annotation Chart, with GO terms found for approximately 60% of all genes in the up- and down-regulated categories of genes. In the “biological processes” category, for up-regulated genes, enrichment of over two-fold was found for GO terms related to protein metabolism, cell-cell signaling, cell adhesion and transport (Figure 4B), whereas for down-regulated genes, it was for DNA and RNA metabolism, cell-cell signaling, cell cycle and proliferation, cell death, cell organization and biogenesis and several others. A comparison of the up- and down-regulated groups showed substantial overrepresentation of genes belonging to RNA and DNA metabolism, cell-cell signaling, cell cycle and proliferation and cell death in the down-regulated group as compared to up-regulated genes (Figure 4B).

Analysis of GO terms for “molecular function” for up-regulated genes showed enrichment of over two-fold for kinase activity, whereas in the down-regulated genes for cytoskeletal activity, nucleic acid binding and enzyme regulator activity (Figure 4C), the latter three groups were significantly overrepresented in the down-regulated gene category.

#### Pathway analysis using KEGG

**Figure 5:**
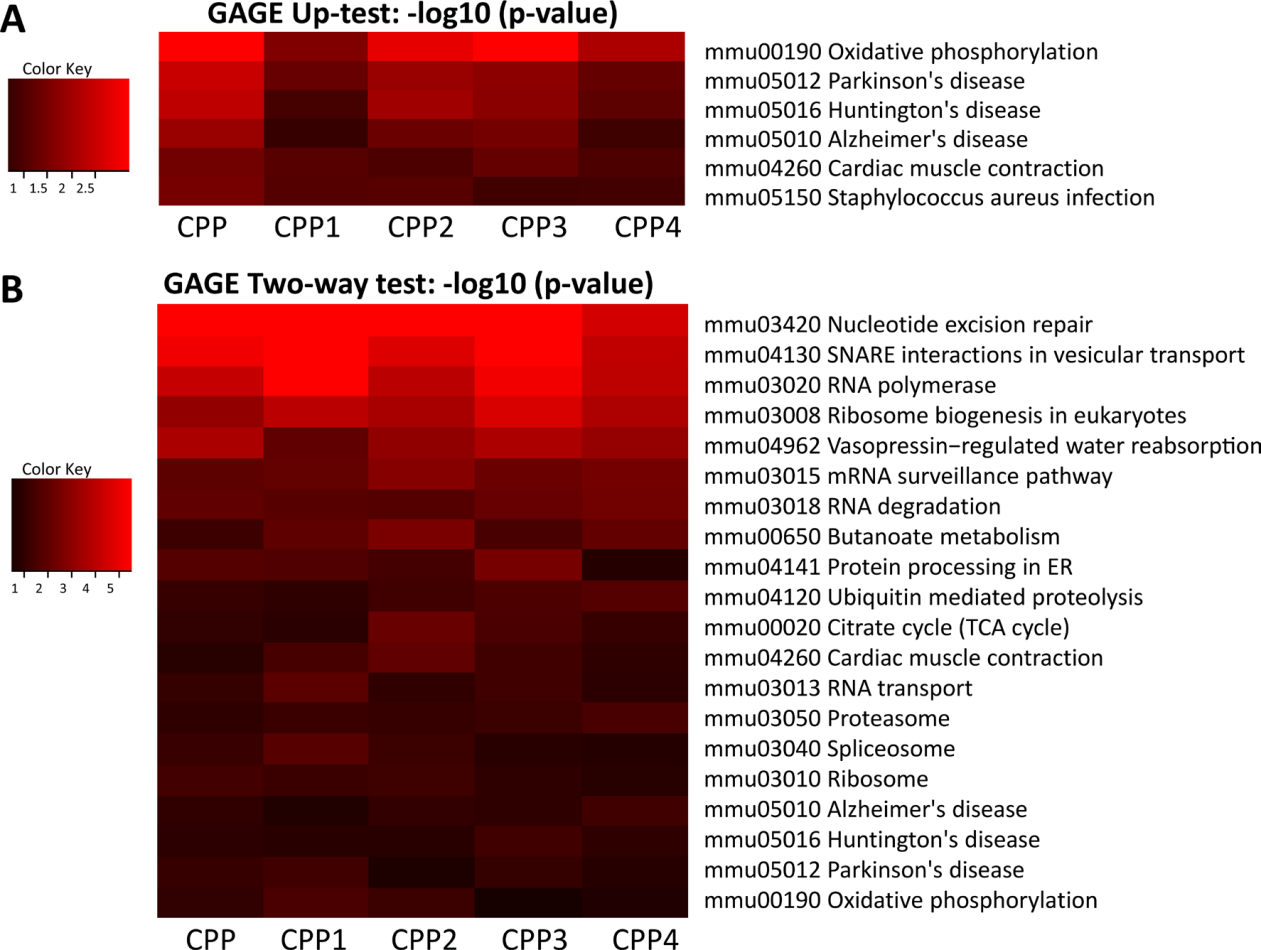
Analysis of pathways deregulated upon parental exposure to CPP (**A**) Heat map of changes in various KEGG pathways obtained after unilateral GAGE analysis. Heat maps (red indicates up-regulation) were generated after unilateral analysis using KEGG pathways and feeding the data for gene expression from five animals (labelled as CPP, CPP1, CPP2, CPP3, CPP4 and CPP5), each individually compared to the averaged control. (**B**) Heat map of changes in various KEGG pathways obtained after bilateral GAGE analysis. Heat maps (red indicates up-regulation) were generated after bilateral analysis using KEGG pathways and feeding the data for gene expression from five animals (labelled as CPP, CPP1, CPP2, CPP3, CPP4 and CPP5), each individually compared to the averaged control.

Unilateral analysis of differentially expressed genes using KEGG software showed the following pathways to be up-regulated in progeny of CPP-exposed animals: oxidative phosphorylation, Alzheimer’s, Parkinson’s and Huntington’s diseases, and cardiac muscle contractions (Figure 5A). Bilateral analysis showed 20 different KEGG pathways up-regulated, including those mentioned above, as well as nucleotide excision repair, RNA polymerase, ribosome biogenesis, mRNA surveillance, RNA degradation, RNA transport and many others (Figure 5B).

A more detailed picture was obtained for differentially expressed genes in 10 different categories, including Alzheimer’s, Parkinson’s and Huntington’s diseases, neurotrophin signaling pathway, mRNA surveillance pathway, oxidative phosphorylation, nucleotide excision repair, spliceosome, proteasome and ribosome function (Supplementary Figures 1–10). For example, cytochrome complexes I, III, IV and V (Cx I, Cx III, Cx IV, Cx V) were up-regulated, whereas cytochrome complex Cx II and *Casp3* were down-regulated in Alzheimer’s, Parkinson’s and Huntington’s diseases (Supplementary Figures 1–3).

The importance of changes in response to CPP was demonstrated by alterations in the neurotrophin signaling pathway (Supplementary Figure 4); progeny of animals exposed to CPP had decreased expression levels of *NRAGE*. NRAGE interacts with the nerve growth factor (NGF) low affinity receptor p75NTR and facilitates cell cycle arrest and NGF-dependent neuronal apoptosis [53]. Expression of calmodulin (CaM) and phosphorylated cAMP-response element binding protein (CREB) decreased in progeny. Disruption of the CaM/CREB pathway could be associated with impaired hippocampal learning and memory [54].

**Figure 6:**
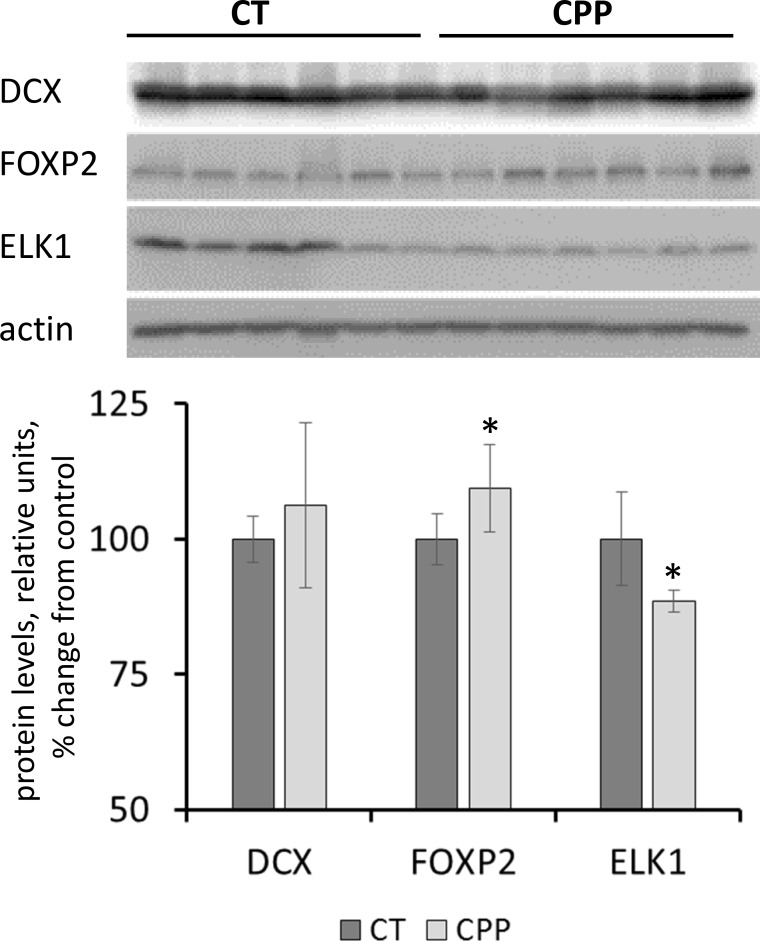
Levels of DCX, FOXP2 and ELK1 in whole brain samples of progeny of CPP-exposed parents Data are shown as average (with SD) arbitrary units of intensity calculated from six individual data points per each experimental group. Due to protein size differences and scarcity of tissue, membranes were re-used several times. Each data point was related to an average intensity of actin samples in a corresponding group and expressed as percent change from controls. Asterisks (^*^) indicate statistically significant (*p* < 0.05) difference to unexposed control, Student's *t*-test.

The expression level of several key proteins involved in neurogenesis and neuroprotection were tested further using western blot analysis. Interestingly, the levels of Dcx protein were unchanged in response to CPP (Figure 6). Doublecortin (DCX) is a cytoskeleton-associated protein that is important for neurogenesis, including migration, axonal guidance and dendrite sprouting [55]. While it was down-regulated on the RNA level, its protein levels were unchanged. This difference may be explained by the compensatory involvement small RNAs or changes in RNA stability and the precise mechanisms of this phenomenon need to be studied in the future. Also, protein levels of neuronal transcription factor FOXP2 were slightly, but significantly (*p <* 0.05) upregulated in the progeny exposed to CPP (Figure 6); FOXP2 is important for the development of brain regions involved in speech and language development [56].

In contrast, the levels of ELK1 protein were decreased in the progeny of animals exposed to CPP (Figure 6). ELK1 belongs to the family of transcription factors abundantly expressed in the nervous system. It is known to participate in neuroprotection against toxic stimuli. Neuroprotective role of ELK1 was demonstrated in mouse model of Hungtington’s disease [57]. It is possible that progeny of CPP-exposed parents has lower potential of neuroprotection in adverse conditions, although this has to be further demonstrated experimentally.

Several components of oxidative phosphorylation machinery were found to be up-regulated, including components of the electron transport chain, NADH dehydrogenases (*Ndufa1*, *Ndufa2*, *Ndufa3*, *Ndufa5*, *Ndufa7*, *Ndufa8*), cytochrome C reductases (*QCR9*, *QCR10*), and cytochrome C oxidases (*COX6A*, *COX6B*, *COX7A*, *COX7B*, *COX17*) (Supplementary Figure 5).

In the nucleotide excision repair pathway, the expression of *PCNA* was reduced (Supplementary Figure 6). This finding is similar to what was found on the protein level – PCNA protein levels were reduced in the BR samples of progeny of CPP-treated animals. The component in the XPC complex, *CETN2*, was also down-regulated in several animals (Supplementary Figure 6).

In the ribosome biogenesis category, many genes encoding ribosomal proteins were found to be up-regulated, including homologs of *L14*, *L23*, *L34e*, *L30e*, *S12*, *S15e*, *S18*, *S26e*, *L44e*, *L27e* and *L38e* (Supplementary Figure 7). Similarly, genes encoding spliceosome Sm proteins were up-regulated in progeny of CPP-exposed animals (Supplementary Figure 8).

Our pioneer study provided the first evidence that paternal exposure to chemotherapy drugs may cause transgenerational changes in the brain of unexposed progeny. Although no DNA damage was observed, changes were found in the levels of DNA repair proteins, cell proliferation and apoptosis, proteins related to DNA methylation, as well as proteins involved in myelin production. All of these proteins are important for brain development and function.

The present analyses present the first study showing that paternal chemotherapy exposure exerts transgenerational effects in the brain tissues of unexposed progeny, albeit to a moderate extent. Paternal chemotherapy exposure led to altered levels of proteins PCNA, BCL2, AKT1, DNMT1, MeCP2, MBP, MYT1L, FOXP2, and ELK1 in FC and BR tissue, although they were often changed in the opposite direction in these two samples. Transcriptomic analysis showed changes only in response to CPP and only in the BR tissues. Further studies are required to determine why this was the only treatment causing changes in the transcriptome. Furthermore, transgenerational changes may be brain-region specific and this specificity needs to be further analyzed in detail, and, most importantly, using larger animal cohorts

Profound changes in neurotrophin pathway and pathways leading to Alzheimer’s, Parkinson’s and Huntington’s diseases suggest that the progeny of CPP-exposed animals may be impaired in various neurological functions and possibly, may even demonstrate altered behavior. Additional molecular, neuroanatomical and behavioral studies are needed to corroborate and extend these pilot results. In the future, it would be important to dissect the cellular changes in the progeny’s brain tissue. It would be prudent to correlate protein levels and cellular changes with global gene expression and, most importantly, to analyze small RNA profiles of the brain tissue of progeny of chemotherapy-exposed parents. It would also be interesting to establish region-specificity of the observed changes.

However, in future research, it would be most important to establish whether changes on the molecular level are paralleled by changes in behavior. The decrease in MYT1L levels may lead to altered cognitive functions. Similarly, the loss of AKT1 may affect neuronal development and, thus, influence a myriad of neuronal functions. At the same time, lower levels of ELK1 may result in lower neuroprotective potential and adverse response to stress in the progeny of CPP-exposed parents. Also, altered MeCP2 levels may be associated with altered chromatin, gene expression and brain anatomy.

Here we used several pathway databases (such as GO, Reactome and KEGG) to establish the degree of involvement of different pathways in the molecular changes in the progeny of exposed parents. While these platforms provide excellent tools to analyse biological repercussions of transgenerational effects, they do not allow quantitative pathway activation analysis. This can be achieved using novel platforms such as the OncoFinder or iPANDA. In the future, we will apply OncoFinder and iPANDA to analyse the pathway activation strength [58, 59]. This approach can also be used to compare transgenerational effects in different organs of the progeny of exposed parents and to correlate gene expression and small RNA expression patterns [60]. Exposures to genotoxic agents can promote and facilitate aging. The observed molecular changes may in turn predispose progeny of exposed parents to accelerated aging. In the future, it would be important to study transgenerational effects in the aging domain [61, 62]. Moreover, in the future it would be important to establish whether changes at the molecular level are parallel by transgenerational changes in behavior and neuroanatomy.

In summation, this study provides a key roadmap for future investigations of the phenomenon of transgenerational effects in the brains of progeny of chemotherapy-exposed parents.

## MATERIALS AND METHODS

### Animal model

In this study, an *in vivo* murine model (8 weeks old male and female BALB/c mice) was used to study the transgenerational effects of chemotherapy drugs. Murine models are widely used, well-characterized and generally accepted for studies of transgenerational effects [63, 64]. The focus of the analysis was on the molecular changes in the frontal cortex (FC) and whole brain (BR) of the progeny. Animals were housed in a pathogen-free facility and given food and water *ad libitum*. The study was approved by the University of Lethbridge Animal Welfare Committee according to guidelines developed by the Canadian Council on Animal Care.

### Treatments

#### Chemotherapy drugs

Based on previous data [7], transgenerational effects of CPP, PCB and MCC were analyzed. CPP is a widely used anticancer drug and immunosuppressive agent which, following metabolic activation, forms DNA adducts [6, 7]. Single doses for anticancer treatment in humans are typically between 400 and 1,200 mg/m2; the equivalent mouse dose for this drug is 130 to 400 mg/kg. Exposing animals to 40–80 mg/kg of CPP resulted in statistically significant, dose-dependent increases in germline mutations in treated animals [6, 7]. A recommended dose of 150 mg/kg of CPP was used in this study. PCB is used to treat a number of cancers, including Hodgkin’s lymphoma. Metabolites of this drug inhibit DNA polymerase and react directly with DNA causing damage [65]. Human single doses do not exceed a maximum of 150 mg/m2; the equivalent mouse dose for this drug is a maximum of 50 mg/kg [6, 7]. In the same study, paternal exposure to clinically relevant doses of PCB (50 mg/kg) also led to significantly increased germline mutation rates [6, 7]. For this research, 13.3 mg/kg of PCB was used.

The streptomycin-derived antibiotic MMC is another well-established anticancer drug. It causes DNA damage, and exposure to single doses of 2.5–5.0 mg/kg leads to pronounced increases in germline mutations in mice [6]. Typical single doses to humans are between 10 and 20 mg/m2, and corresponding mouse doses are 3–6 mg/kg. To evaluate the transgenerational effects of paternal exposure to MMC, a recommended dose of 5 mg/kg [6] was chosen.

#### Husbandry

Four male BALB/c mice were exposed at eight weeks of age to PCB, CPP or MMC. Eight weeks after exposure, the chemotherapy-treated males were mated with untreated females (2 females/1 male). Based on the timing of mouse spermatogenesis, such mating would reveal spermatogonial effects. The litter size in MMC treatment groups appeared to be smaller than others (only 9 pups). Other groups had over 20 pups. Eight pups/group were used in the analysis. The progeny were obtained and sacrificed at the age of one week. To avoid data bias, animals from different litters were evenly distributed among four treatment groups. Upon sacrificing, the brains were dissected to separate the FC from the rest of the brain. Due to the small size of the brains, further dissections were not possible.

The FC and the rest of the brain (BR) were frozen and stored in −80°C for further analysis.

### Protein extraction and western immunoblotting

FC and BR tissues were used for Western immunoblotting analysis to determine the levels of various proteins as described previously [66]. Briefly, tissue samples were sonicated in 0.4 ml of ice-chilled 1% sodium dodecyl sulphate (SDS) and immediately boiled for 10 minutes. Protein concentrations were determined using Bradford Assay (BioRad, Hercules, CA, USA). Protein extracts were separated by SDS–polyacrylamide electrophoresis (PAGE) in slab gels of 12% polyacrylamide, and transferred to Hybond-P PVDF membranes (Amersham, Baie d’Urfe, QC, Canada). Membranes were incubated with antibodies against fifteen proteins. These were γH2AX, ELK1, AKT1, MeCP2, (1:1000, Cell Signaling, Danvers, MA, USA), KU70, p 53, BCL2, PCNA, FOXP2, LYNX1, DCX (1:1000, Santa Cruz Biotechnology), MBP (1:500, Millipore, Billerica, MA, USA), and DNMT1, MYT1L, actin (1:1000, Abcam, Cambridge, MA). Due to protein size differences and scarcity of tissue, membranes were re-used and re-probed several times. Antibody binding was revealed by incubation with horseradish peroxidase-conjugated secondary antibodies (Amersham) and ECL Plus immunoblotting detection (Amersham). Chemiluminescence was detected by a FluorChem HD2 System (Cell Biosciences/ProteinSimple, Santa Clara, CA, USA). Unaltered PVDF membranes were stained with Coomassie Blue (BioRad).

### RNA extraction and transcriptome profiling

Total RNA was isolated using TRIzol Reagent (GE Healthcare Life Sciences, Buckinghamshire, UK). RNA samples were quantified using ultraviolet spectroscopy (NanoDrop, Wilmington, DE) and handed over to the Gene Expression Facility for further transcriptome profiling. The RNA was quantified and found to be of a high quality using a Bioanalyser 2100 (Agilent, CA, USA). RNA labeling and microarray hybridization were performed by the Lethbridge Epigenetics Laboratory Illumina Facility. MouseRef-8 v2.0 Expression BeadChip whole-genome expression arrays (Illumina, CA, USA) were used in this study. Each array on the MouseRef-8 v2.0 Expression BeadChip targets 25,600 well-annotated RefSeq transcripts, over 19,100 unique genes derived from the National Center for Biotechnology Information Reference Sequence (NCBI) and other sources (Illumina). In total, 40 animals were used, five animals per each treatment (CPP, MMC, PCB) and one control per each of two brain regions.

In brief, each RNA sample was amplified using the Ambion Illumina RNA amplification kit with biotin UTP (Enzo, ON, Canada) labeling. The Ambion Illumina RNA amplification kit uses T7 oligo(dT) primer to generate single-stranded cDNA followed by a second strand synthesis to generate double-stranded cDNA, which is then column purified. In vitro transcription was conducted to synthesize biotin-labeled cRNA using T7 RNA polymerase. The cRNA was column purified and checked for size and yield. cRNA was hybridized using standard Illumina protocols with streptavidin-Cy3 (Amersham, IL, USA). Arrays were scanned on an Illumina BeadStation and analyzed using BeadStudio (Illumina) as recommended by the manufacturer. Normalization, clustering and significance analysis were done by the Lethbridge Epigenetics Laboratory Illumina Facility as previously described [67, 68].

Data were normalized and analyzed using Illumina BeadStudio Software. The false discovery rate (FDR) was controlled using the Benjamini-Hochberg method. The Illumina Custom Model took the FDR into account and was used to analyze the data. Differential gene expression from control cells was determined to be statistically significant if the *p*-value was lower than 0.05 after the Benjamini-Hochberg method adjustment.

### Bioinformatics analysis

#### Reactome and GO term enrichment analysis

Pathway information for the differentially expressed genes was retrieved from the Reactome database (http://www.reactome.org). Pathway enrichment analysis was performed by comparing the number of genes included in the pathways for up- or down-regulated genes, after normalizing them against genes in the database. In addition to pathway annotations using the Reactome database, additional annotations with gene ontology (GO) terms were performed using the Database for Annotation, Visualization and Integrated Discovery (DAVID v6.7, http://david.abcc.ncifcrf.gov). GO term enrichments were calculated by dividing the ratio between genes found for a specific term and all genes in the up- or down-regulated set by the ratio between all known mouse genes for a specific term and all known mouse genes:

E = N GOt/ Nt: N GOt hg/Nt hg, where E = enrichment; N GOt = number of genes assigned to a specific GO term among the up- or down-regulated genes in CPP treatment group; Nt = total number of up- or down-regulated genes in the group; N GOt hg = number of genes assigned to a specific GO term in the entire mouse genome; Nt hg = total number of genes in the mouse genome.

Genes belonging to specific GO terms were than clustered according to DAVID clustering of annotation terms using the Functional Annotation Clustering tool for the following categories: molecular function and biological process.

#### Gene set analysis (GSA) using KEGG pathways

The Generally Applicable Gene-set Enrichment (GAGE) method was used as a GSA as previously published [69]. We used the KEGG pathways gene set for comparative analysis. The data for the control group of genes were averaged across five samples and each individual sample in the CPP group was analyzed against the averaged control. The mean log fold change of the genes in the specific KEGG pathway was compared to the mean log fold change of all of the genes on the array. First, a unidirectional analysis for the up- or down-regulated genes was performed, and the up- and down-regulated pathways were identified. Next, a bidirectional analysis was performed, disregarding the direction of the change in gene expression, and the up- or down-regulated pathways were obtained that contained both up- and down-regulated genes. The heat maps were than generated using the FDR adjusted *p*-value of < 0.05. Specific KEGG pathways show data for the expression of individual genes in all five animals in the CPP group related to the averaged control (Supplementary Figures 1–10).

## SUPPLEMENTARY MATERIALS FIGURES


