# Supplementary material for: Adverse effects of paternal chemotherapy exposure on the progeny brain: intergenerational chemobrain
Source: Oncotarget. 2018 Jan 23;9(11):10069–82. doi: 10.18632/oncotarget.24311 (PMC5839372; doi:10.18632/oncotarget.24311)
Supplement: Supplementary file 1 [file oncotarget-09-10069-s001.pdf]

Adverse effects of paternal chemotherapy exposure on the progeny brain: intergenerational chemobrain

SUPPLEMENTARY MATERIALS

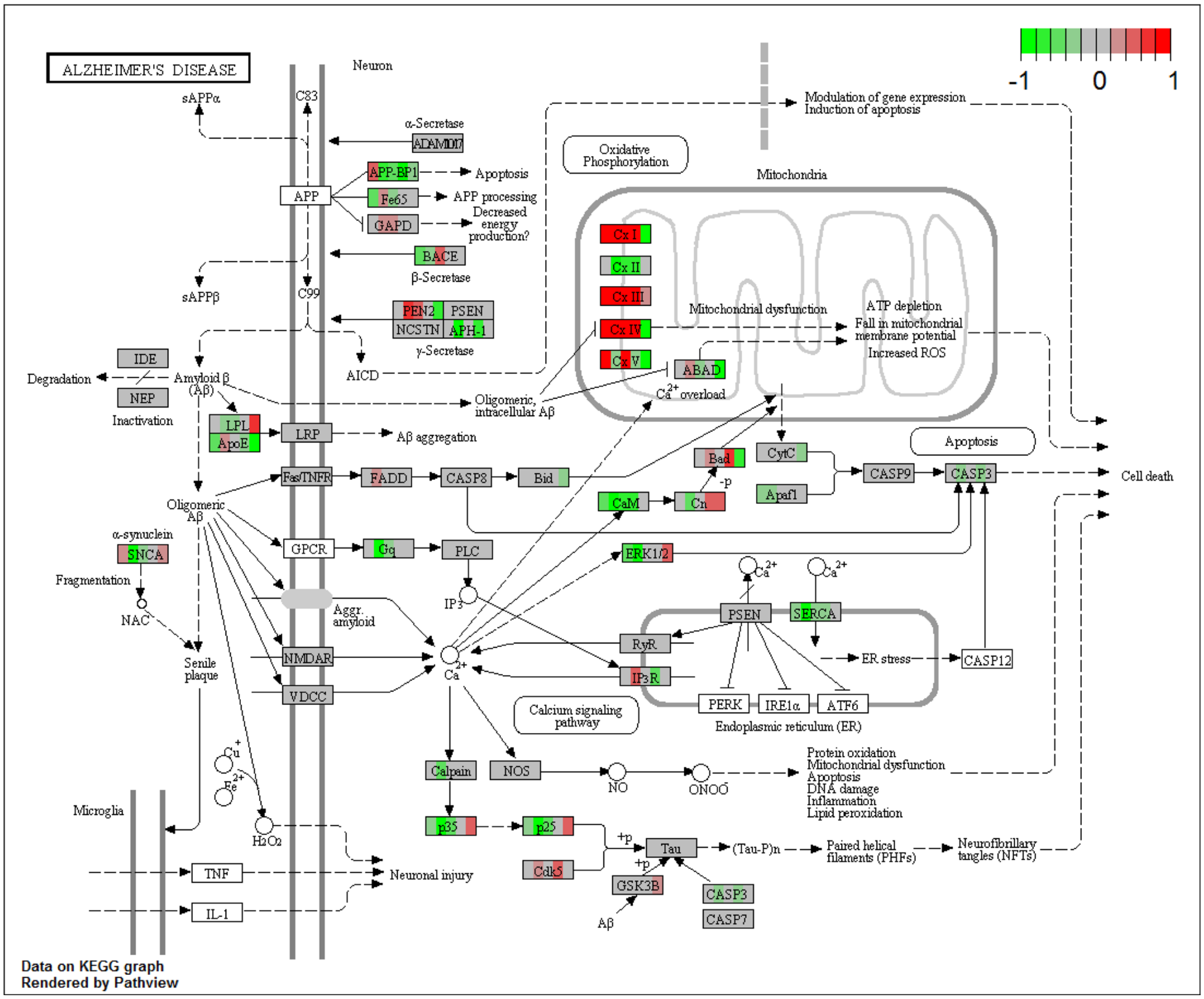

Supplementary Figure 1: KEGG/Pathway analysis – Alzheimer's disease pathway.

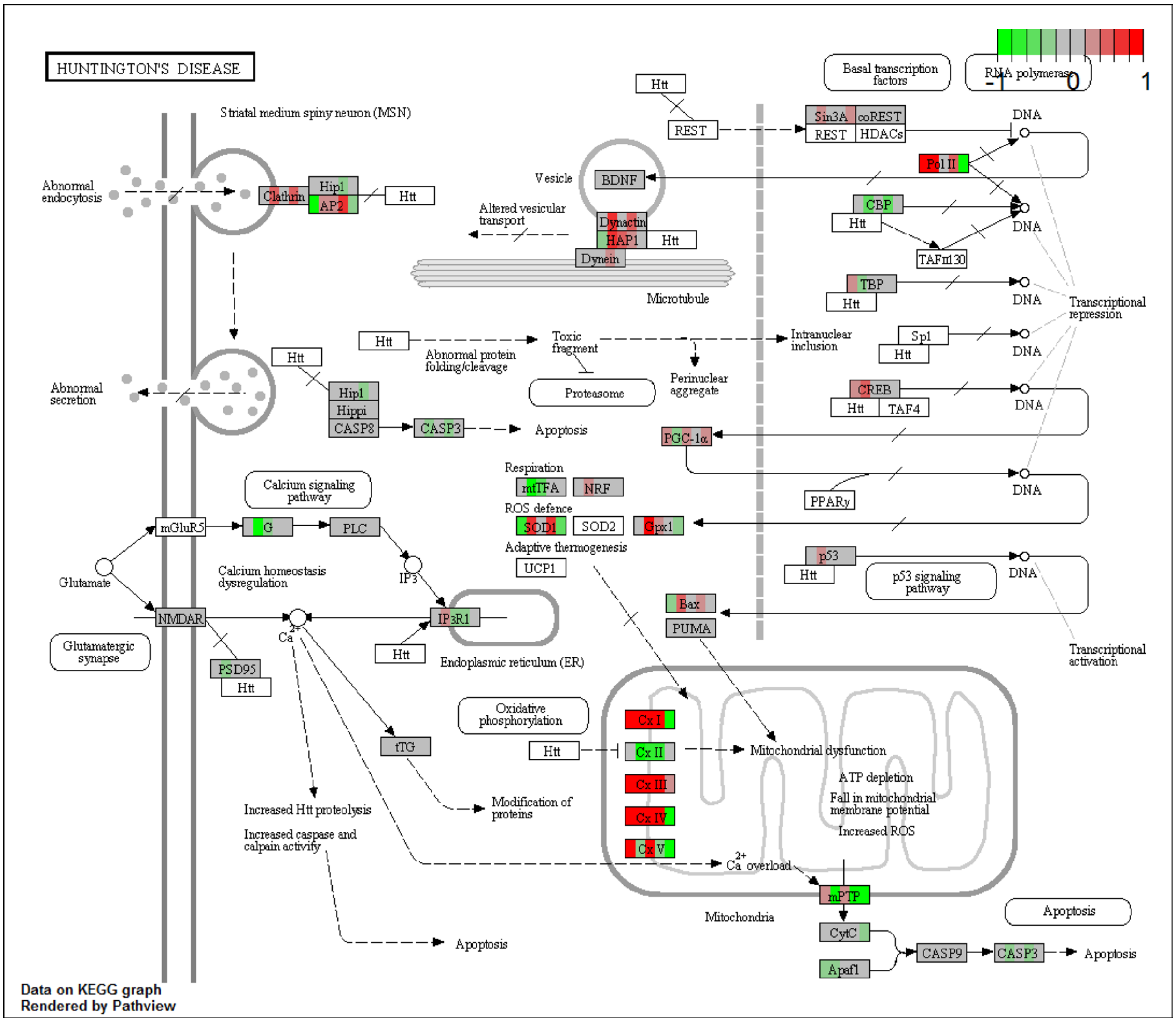

Supplementary Figure 2: KEGG/Pathway analysis – Huntington's disease pathway.

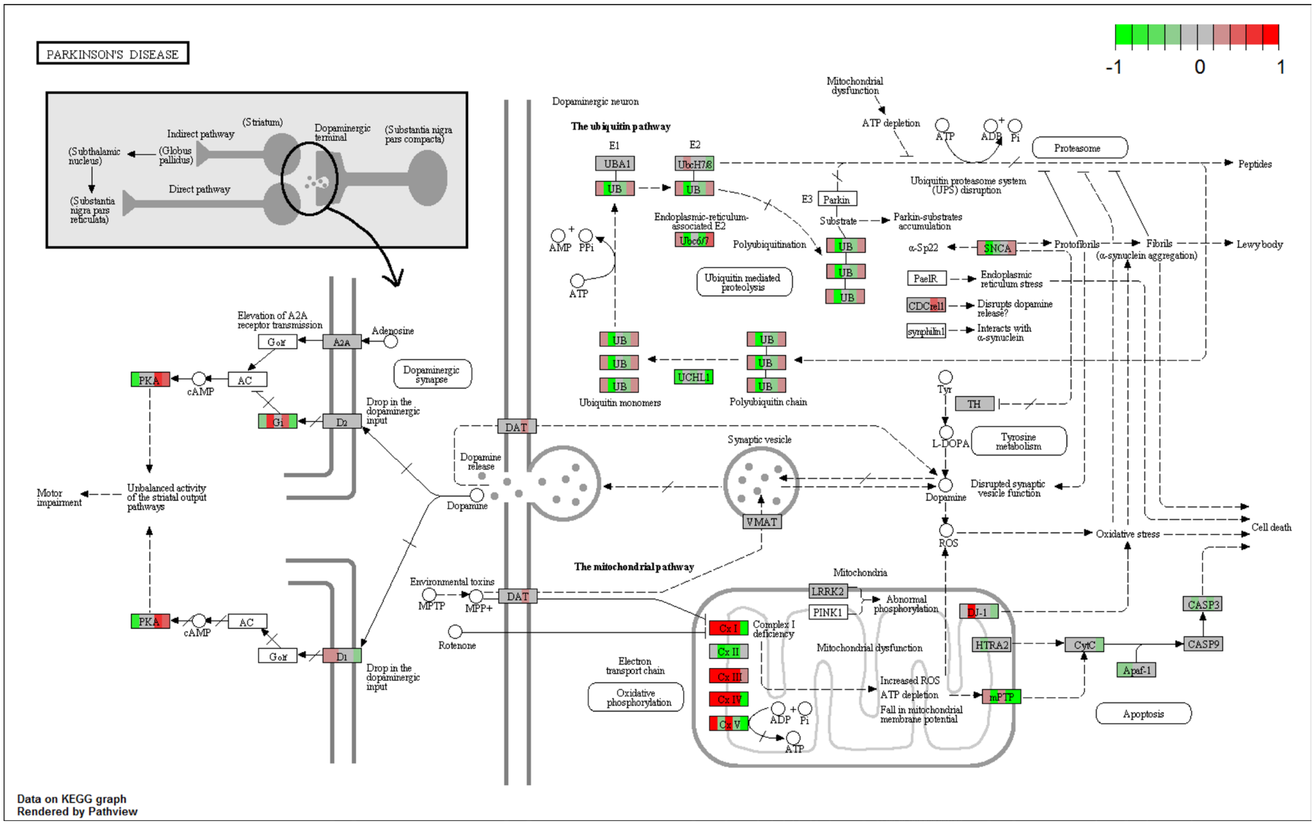

Supplementary Figure 3: KEGG/Pathway analysis – Parkinson's disease pathway.

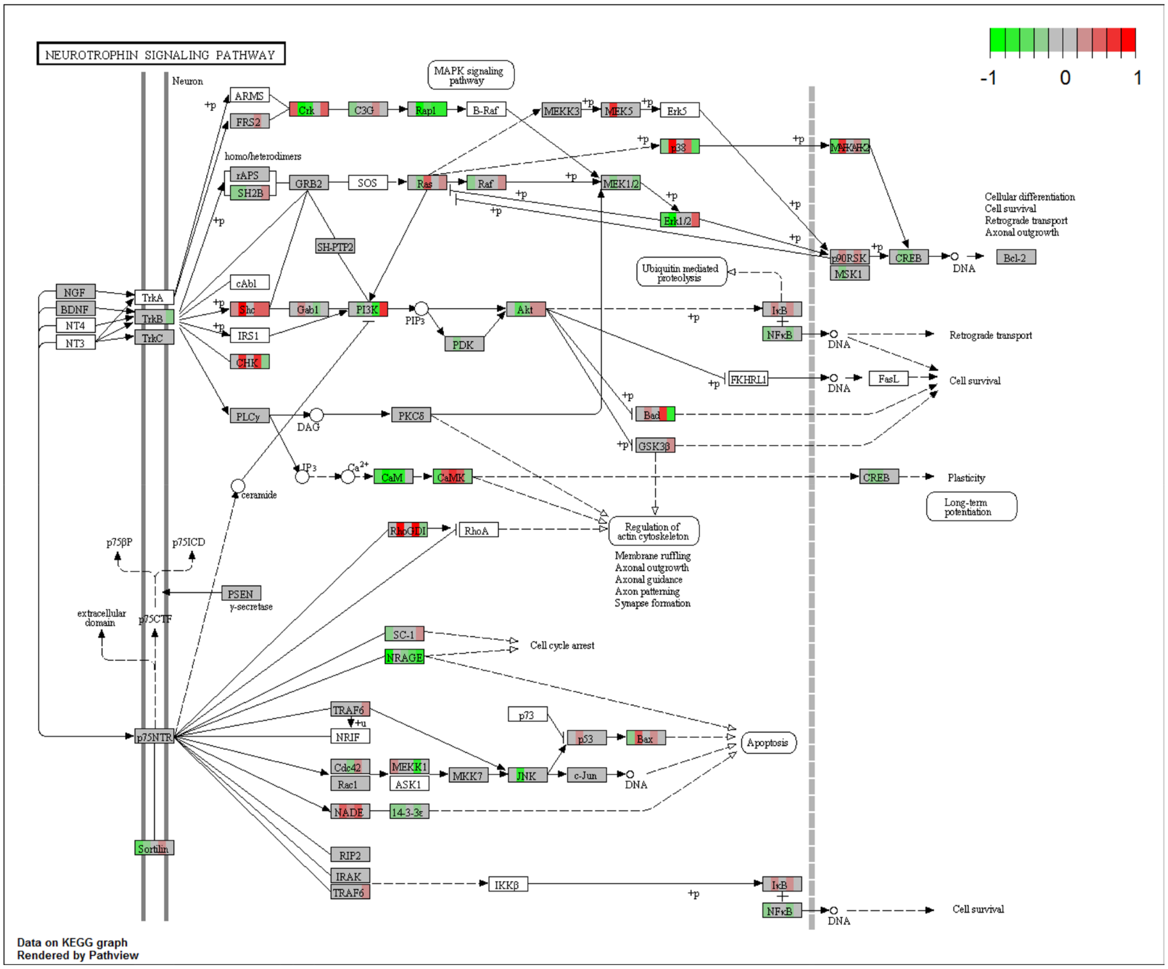

Supplementary Figure 4: KEGG/Pathway analysis – Neurotrophin signalling pathway.

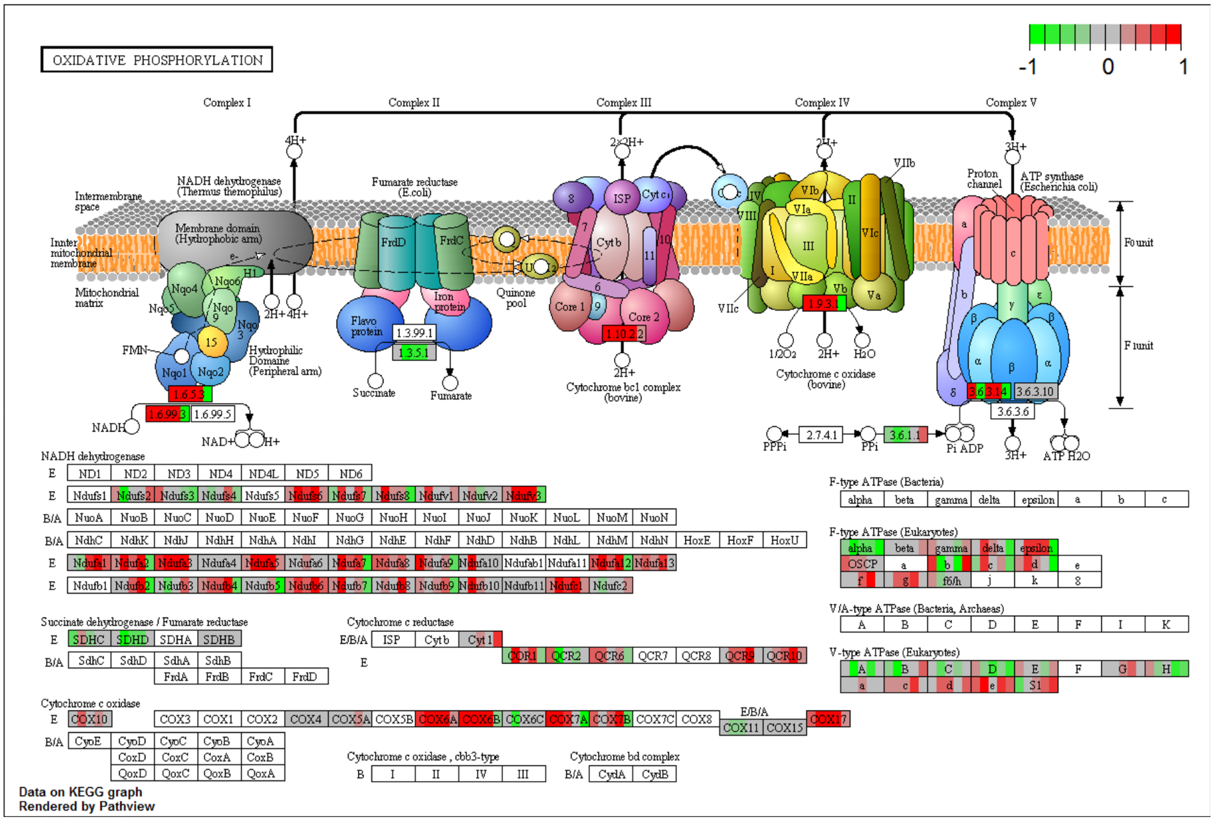

Supplementary Figure 5: KEGG/Pathway analysis - Oxidative phosphorylation pathway.

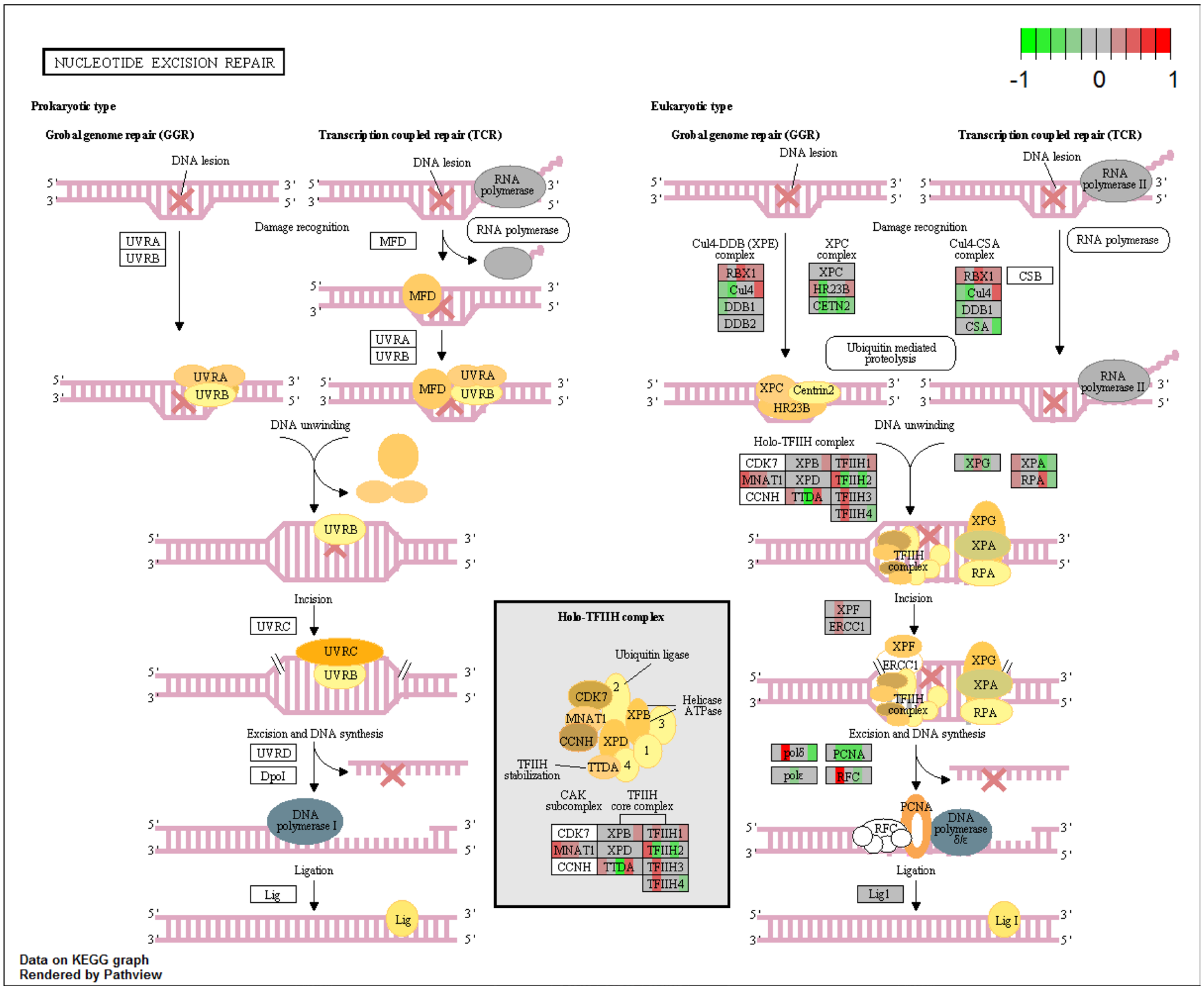

Supplementary Figure 6: KEGG/Pathway analysis - Nucleotide excision repair.

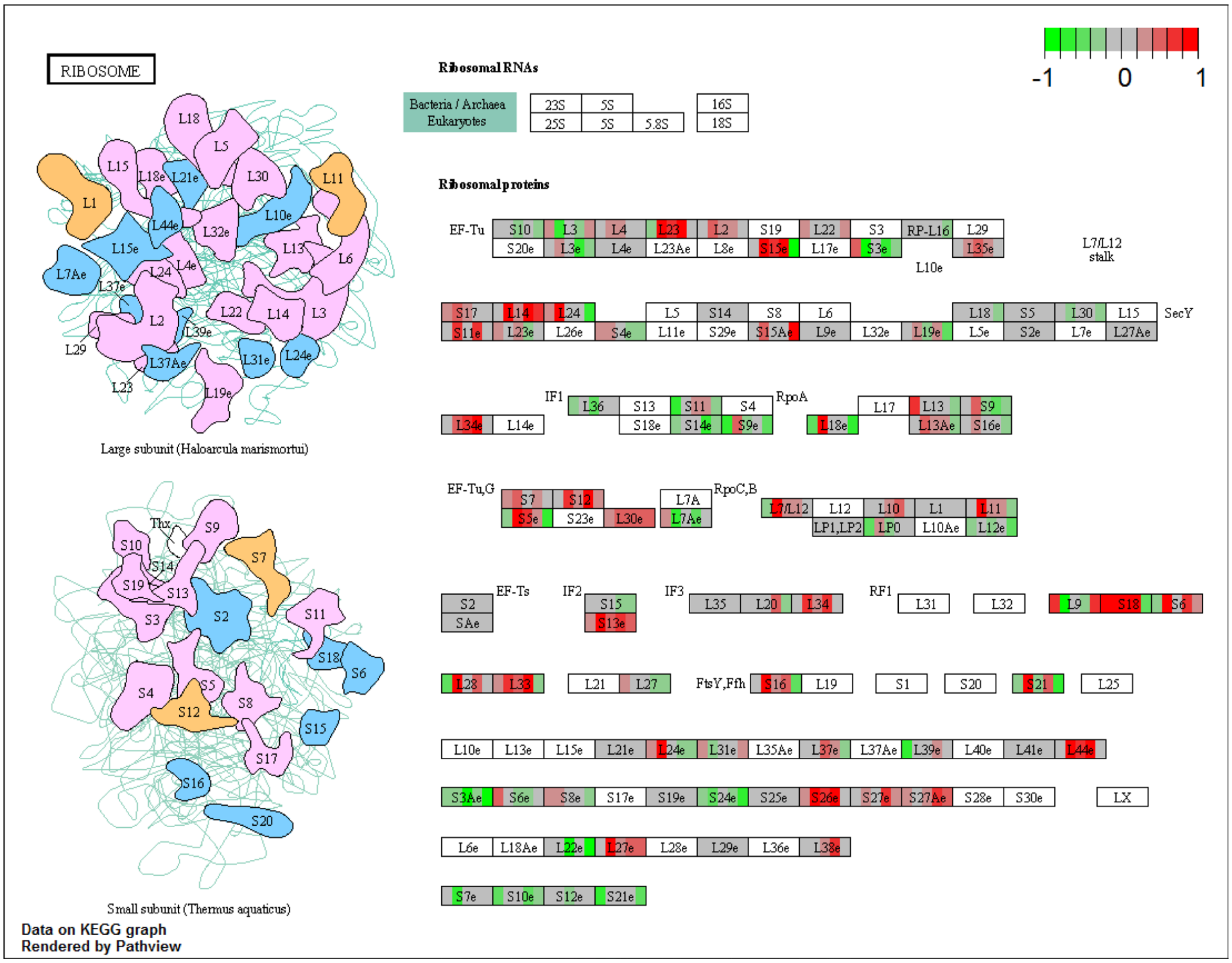

Supplementary Figure 7: KEGG/Pathway analysis - Ribosome.

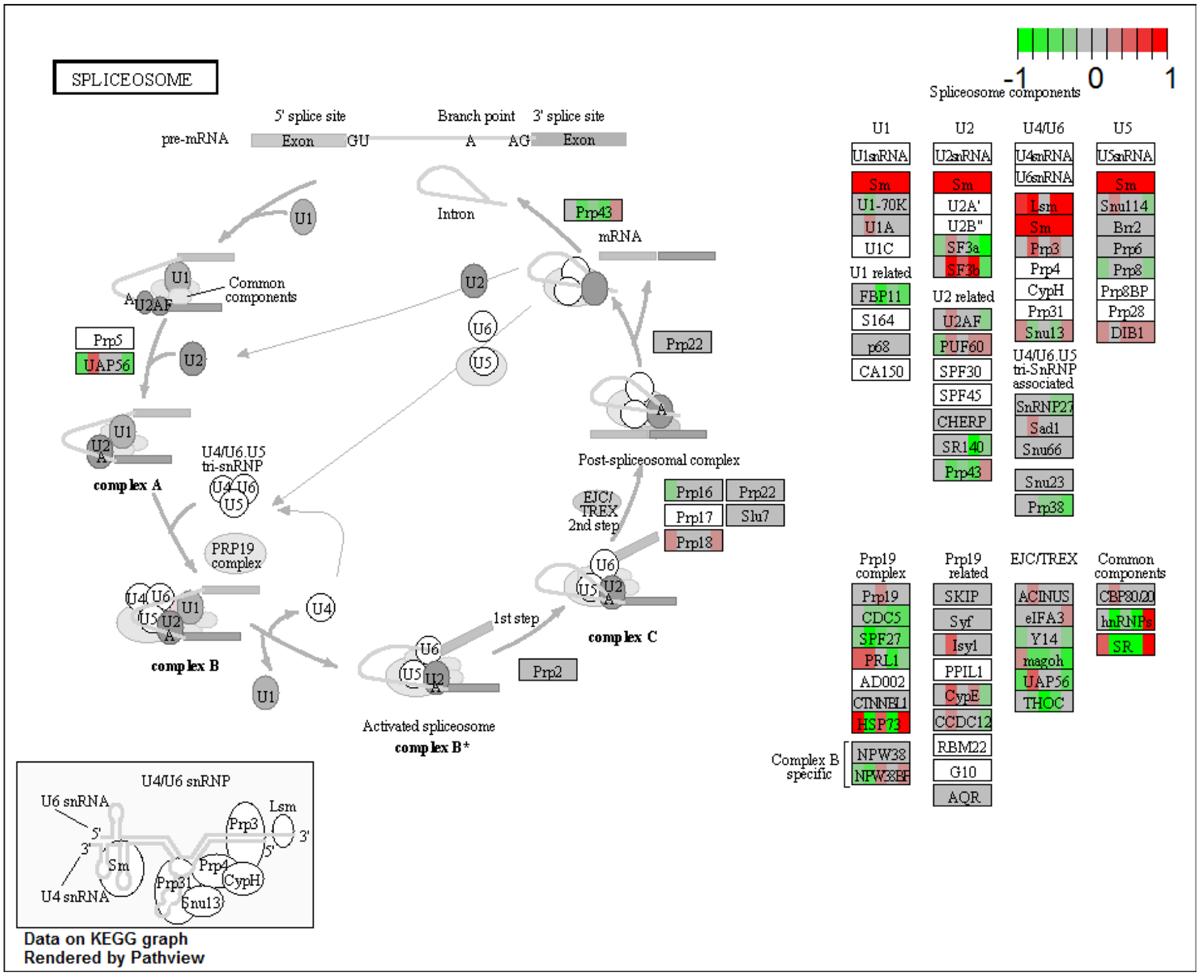

Supplementary Figure 8: KEGG/Pathway analysis - Spliceosome.

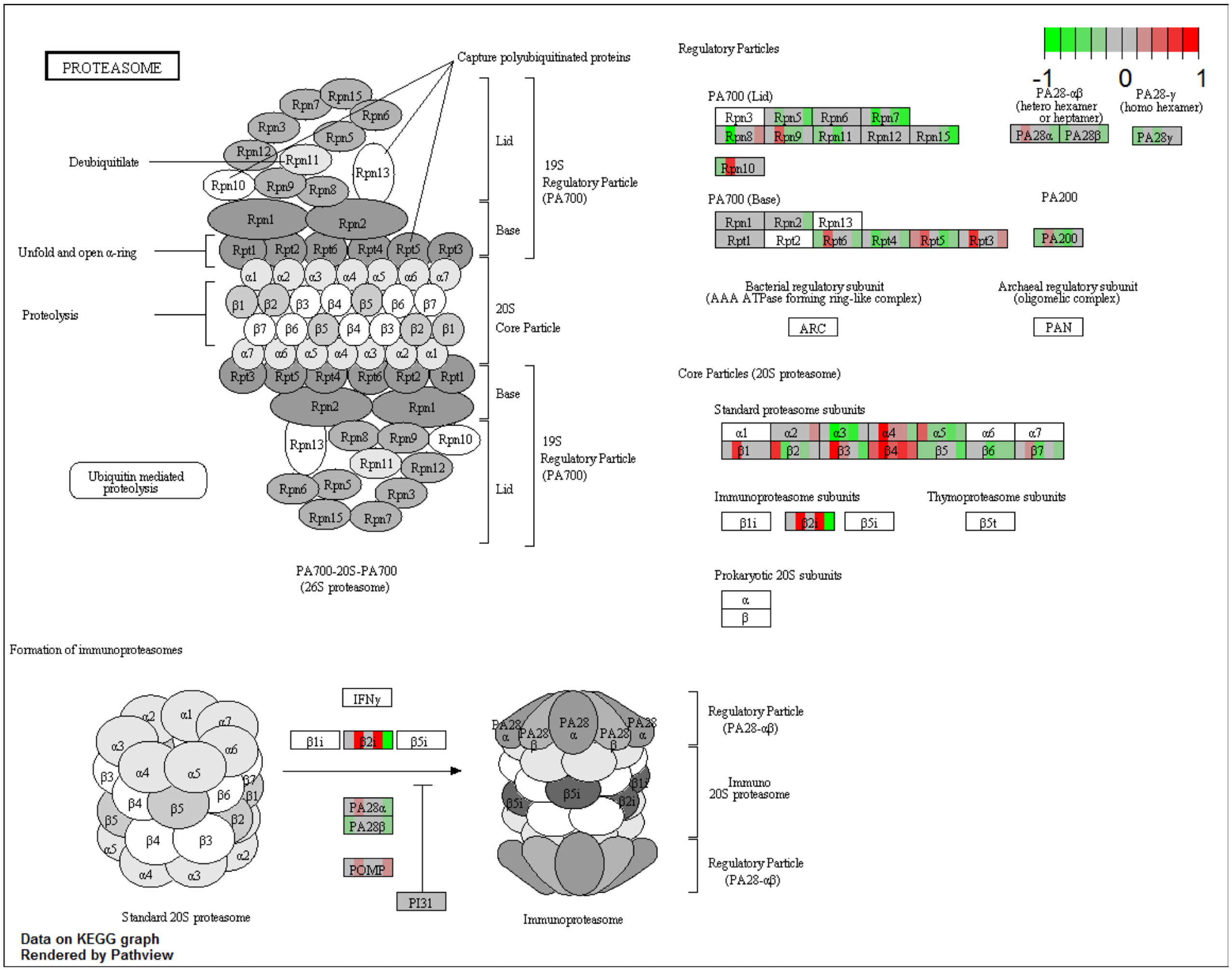

Supplementary Figure 9: KEGG/Pathway analysis - Proteasome.

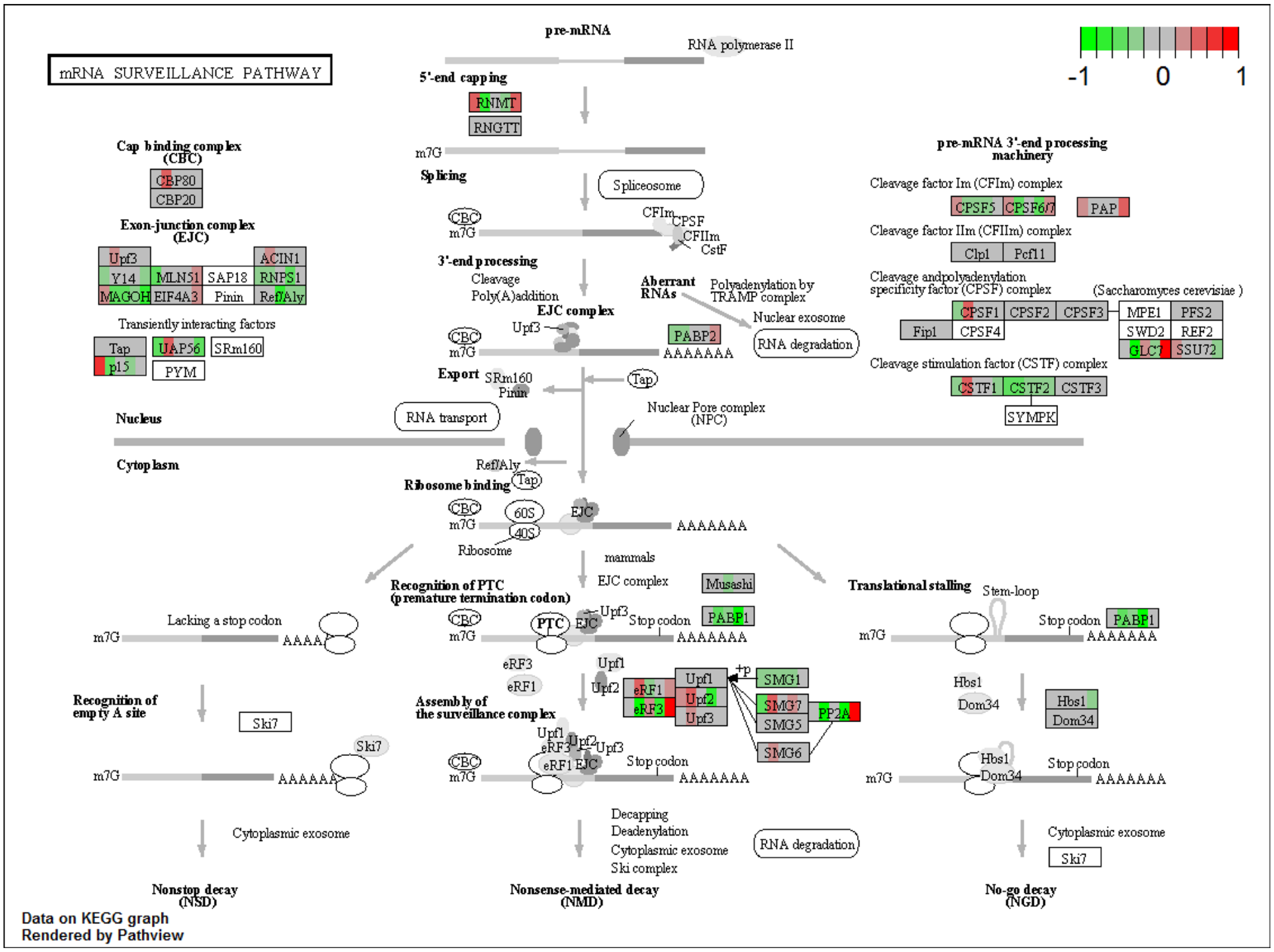

Supplementary Figure 10: KEGG/Pathway analysis - mRNA surveillance pathway.
